# Endothelial Damage, Neutrophil Extracellular Traps and Platelet Activation in COVID-19 vs. Community-Acquired Pneumonia: A Case–Control Study

**DOI:** 10.3390/ijms241713194

**Published:** 2023-08-25

**Authors:** Paula González-Jiménez, Raúl Méndez, Ana Latorre, Noé Mengot, Mónica Piqueras, Soledad Reyes, Antonio Moscardó, Ricardo Alonso, Isabel Amara-Elori, Rosario Menéndez

**Affiliations:** 1 Pneumology Department, La Fe University and Polytechnic Hospital, 46026 Valencia, Spain; paulagonzalezjimenez@gmail.com (P.G.-J.); noemen96@gmail.com (N.M.); solreyes07@gmail.com (S.R.); milisaelori@hotmail.com (I.A.-E.); rosmenend@gmail.com (R.M.); 2 Respiratory Infections, Health Research Institute La Fe (IISLAFE), 46026 Valencia, Spain; latorrecampos.am@gmail.com; 3 Medicine Department, University of Valencia, 46010 Valencia, Spain; piqueras_mon@gva.es; 4 Center for Biomedical Research Network in Respiratory Diseases (CIBERES), 28029 Madrid, Spain; 5 Laboratory Department, La Fe University and Polytechnic Hospital, 46026 Valencia, Spain; alonso_ricdia@gva.es; 6 Hemostasis and Thrombosis Unit, La Fe University and Polytechnic Hospital, 46026 Valencia, Spain; moscardo_ant@gva.es

**Keywords:** COVID-19, CAP, case–control, endothelial damage, platelet activation, neutrophil extracellular traps

## Abstract

COVID-19 has been a diagnostic and therapeutic challenge. It has marked a paradigm shift when considering other types of pneumonia etiology. We analyzed the biomarkers related to endothelial damage and immunothrombosis in COVID-19 in comparison to community-acquired pneumonia (CAP) through a case–control study of 358 patients with pneumonia (179 hospitalized with COVID-19 vs. 179 matched hospitalized with CAP). Endothelial damage markers (endothelin and proadrenomedullin), neutrophil extracellular traps (NETs) (citrullinated-3 histone, cell-free DNA), and platelet activation (soluble P-selectin) were measured. In-hospital and 1-year follow-up outcomes were evaluated. Endothelial damage, platelet activation, and NET biomarkers are significantly higher in CAP compared to COVID-19. In-hospital mortality in COVID-19 was higher compared to CAP whereas 1-year mortality and cardiovascular complications were higher in CAP. In the univariate analysis (OR 95% CIs), proADM and endothelin were associated with in-hospital mortality (proADM: CAP 3.210 [1.698–6.070], COVID-19 8.977 [3.413–23.609]; endothelin: CAP 1.014 [1.006–1.022], COVID-19 1.024 [1.014–1.034]), in-hospital CVE (proADM: CAP 1.623 [1.080–2.439], COVID-19 2.146 [1.186–3.882]; endothelin: CAP 1.005 [1.000–1.010], COVID-19 1.010 [1.003–1.018]), and 1-year mortality (proADM: CAP 2.590 [1.644–4.080], COVID-19 13.562 [4.872–37.751]; endothelin: CAP 1.008 [1.003–1.013], COVID-19 1.026 [1.016–1.037]). In conclusion, COVID-19 and CAP showed different expressions of endothelial damage and NETs. ProADM and endothelin are associated with short- and long-term mortality.

## 1. Introduction

The emergence of SARS-CoV-2 infection in December 2019 has had repercussions on a global economic, social, and health scale. The clinical manifestations that have constituted the COVID-19 disease represent a wide range of clinical scenarios—from asymptomatic infections to severe cases with fatal outcomes [1]. The pathophysiological mechanisms behind this disease are intricate. They are determined by both the microorganism (virulence, viral load, etc.) and the host response (inflammatory response, innate immune response, and acquired immune response) [2]. Understanding such mechanisms is, therefore, key to COVID-19 prevention, management, and treatment.

Endothelial damage—such as degradation and denudation of glycocalyx, the disintegration of intercellular junctions, and endothelial cell death leading to a procoagulant state—and platelet activation are pathophysiological processes present in sepsis [3,4] and related to poorer outcomes. In SARS-CoV-2 pneumonia, a higher proportion of thrombus complications compared to bacterial pneumonia has been observed [5,6]. Some studies have also reported endothelial damage and platelet activation [7].

Neutrophil extracellular traps (NETs) are an innate immunity mechanism that traps and kills microorganisms, as described in 2004 by Brinkmann et al. [8,9]. Neutrophil activation is mainly carried out by microorganisms, host inflammatory response, or platelet activation [10,11]. Both an excess and deficiency thereof have shown deleterious effects. This double-edged sword marks complexity within immunology [12]. In a post hoc analysis of a randomized clinical trial (RCT) in patients with CAP, Ebrahimi et al. found that high initial serum levels of nucleosomes (as a marker of NETosis) predict longer time to clinical stability, longer hospital stays, and a higher risk of 30-day mortality [13]. In COVID-19 pneumonia, there are some studies that analyzed NETosis compared to healthy controls [14,15].

To date, different clinical features have been identified between SARS-CoV-2 pneumonia and CAP [16] including vulnerable populations and innate inflammatory response. Nevertheless, no case–control studies have compared SARS-CoV-2 pneumonia and CAP as they relate to biomarkers that mirror endothelial damage, NET formation, and platelet activation. We hypothesized that there could be differences in NETs, endothelial damage, and platelet activation between CAP and COVID-19 pneumonia—even after matching populations—measurable by systemic biomarkers.

Thus, we aimed to analyze endothelial biomarkers (endothelin and proadrenomedullin (proADM)), NETs (cell-free DNA (cfDNA) and citrullinated histone H3 (CitH3)), and platelet activation (soluble P-selectin (sP-selectin)) during the acute phase of SARS-CoV-2 pneumonia and compare such findings with a matched historical cohort of CAP patients. We also aimed to explore their association with clinical outcomes.

## 2. Results

### 2.1. Patient Characteristics

The demographic characteristics and comorbidities of enrolled patients with SARS-CoV-2 pneumonia and CAP after matching are reported in Table 1. The matched cohorts comprised 179 patients in each group. The demographic characteristics and comorbidities of the initial CAP cohort (N = 1115) are reported in Supplemental Table S1. After matching was performed with the MatchIt package [17], which allows for the pairing of both cohorts, the median patient ages were 65 (53–79) and 70 years (56–79) in COVID-19 and CAP, respectively. Around 60% of patients were male in both groups. Furthermore, 68.8% of patients with CAP were current or former smokers; in the COVID-19 group, the figure was 26.8%.

A higher percentage of patients with overweight, asthma, or chronic obstructive pulmonary disease (COPD) was observed in the COVID-19 group. Patients with CAP had more chronic heart disease. Similar rates of renal chronic disease or neurological disease were observed in both groups (15.1% vs. 14.5% and 15.1% vs. 11.7%, respectively). Pneumonia infiltrates were bilateral in 62% and 18.4% of the COVID-19 and CAP groups, respectively. Maximum respiratory support is detailed in Table 1, according to pneumonia etiology. Briefly, patients with COVID-19 required more respiratory support than those with CAP.

### 2.2. Clinical Outcomes: In-Hospital and 1-Year Follow-Up Complications

In-hospital and total 1-year (in-hospital and extra-hospital) mortality and complications differences between groups are described in Table 2. In patients with SARS-CoV-2 pneumonia, there was higher in-hospital mortality compared to CAP (14.5% vs. 3.9%; *p* = 0.001), although there were no statistically significant differences in cardiac complications except for pulmonary embolism (5.0% vs. 0.0%; *p* = 0.002, respectively). In 1-year outcomes from admission, we observed cardiovascular complications more frequently, such as arrhythmia, congestive heart failure, and stroke complications in patients with CAP. Nevertheless, more cases of pulmonary embolism were reported in patients with SARS-CoV-2. No statistically significant differences were found in either mortality or acute coronary syndrome as a complication.

Clinical outcomes were also evaluated separately in the long term (in-hospital survivors). Table 3 shows outcomes from hospital discharge to 1-year follow-up. Patients with CAP had higher 1-year mortality and cardiovascular complications compared to those with COVID-19.

### 2.3. Endothelial Damage, NETosis, Platelet Activation, and Inflammatory and Immunological Markers

**Figure 1:**
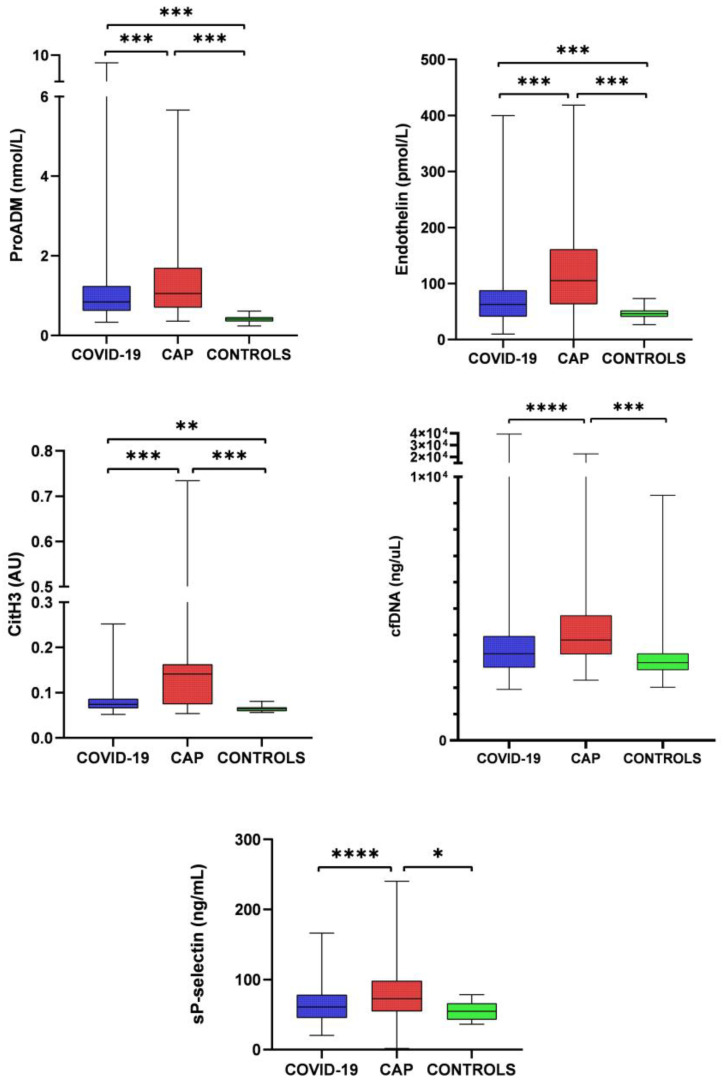
NETosis, platelet activation, and endothelial damage in SARS-CoV-2 pneumonia and CAP. Boxes represent interquartile ranges and whiskers, minimum to maximum values. ProADM, proadrenomedullin; cfDNA, cell-free DNA; citH3, citrullinated histone H3. * *p* < 0.05, ** *p* < 0.01, *** *p* < 0.001, **** *p* < 0.0001.

Differences in endothelial biomarkers, NETs, and platelet activation between patients with COVID-19 pneumonia and those with CAP are reported in Figure 1. Endothelial damage, NET biomarkers, and platelet activation are significantly higher in the latter group.

**Figure 2:**
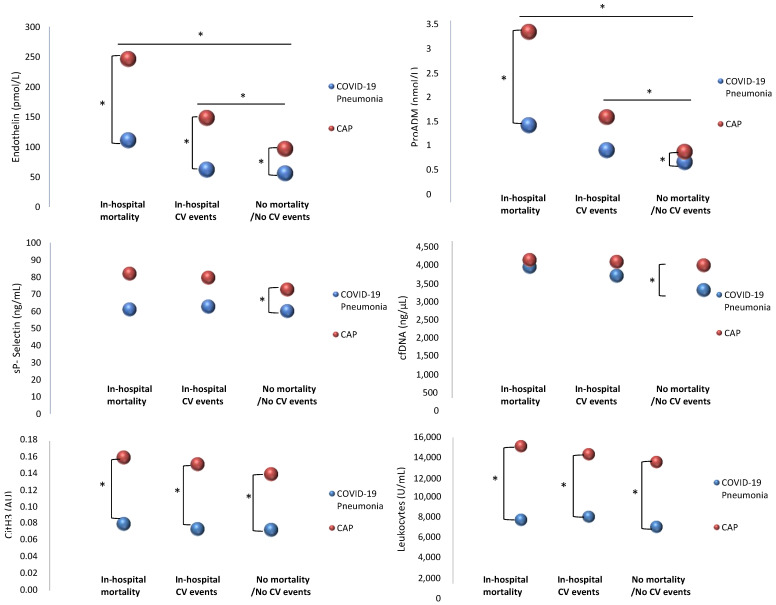
Comparison between NETosis, endothelial damage, platelet activation, and in-hospital complications in COVID-19 and CAP. The spheres represent the median values. * *p* < 0.05.

Differences in biomarker levels between CAP and COVID-19 pneumonia in patients who present in-hospital mortality, in-hospital cardiovascular events (CVE), or none of these events are represented in Figure 2. Significantly higher levels of endothelin and proADM were found in patients with in-hospital mortality and those with CVE; however, levels were more elevated in CAP patients compared to those with COVID-19 pneumonia. Patients with CAP who died during hospitalization had significantly higher levels of endothelial damage biomarkers (endothelin 246.20 [233.19, 296.60] vs. 110.80 [70.46, 194.70] pmol/L, *p* < 0.001; proADM 3.28 [2.60, 4.42] vs. 1.48 [1.22, 1.89] nmol/L, *p* = 0.010), CitH3 (0.160 [0.079, 0.183] vs. 0.080 [0.067, 0.095] AU, *p* = 0.009), and leukocytes (15,030 [7050, 28,110] vs. 6955 [5017, 9593] U/mL, *p* = 0.008), compared to patients with COVID-19. Patients with CAP who presented cardiovascular events during hospitalization had significantly higher levels of endothelin (148.10 [104.18, 213.61] vs. 62.05 [29.11, 194.58] pmol/L, *p* = 0.030), CitH3 (0.152 [0.071, 0.166] vs. 0.074 [0.062, 0.081] AU, *p* = 0.026), and leukocytes (14,150 [11,930, 15,950] vs. 7300 [5480, 9300] U/mL, *p* < 0.001), compared to patients with COVID-19. Patients with CAP who did not die or present CVE showed significantly higher levels of all the studied biomarkers compared to COVID-19.

**Figure 3:**
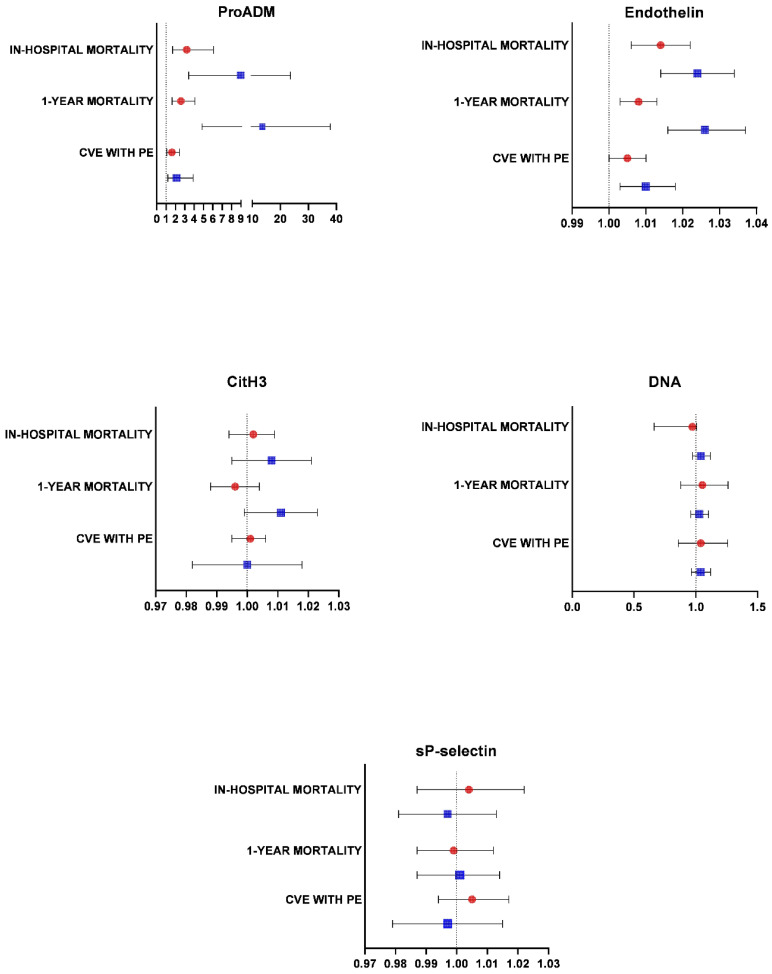
Results of biomarkers expressed in OR and outcomes. Circles and boxes represent odds ratios and whiskers, at 95% confidence intervals. Blue and red colors represent COVID-19 and CAP, respectively.

ProADM and endothelin were associated with in-hospital mortality, in-hospital CVE, and 1-year mortality from admission in both cohorts (COVID-19 and CAP), expressed in odds ratio (OR) and 95% CI (Figure 3).

## 3. Discussion

The main findings of the study are as follows: (1) After matching, patients with CAP have higher levels of endothelial biomarkers (proADM and endothelin), NETs (cfDNA and CitH3), and platelet activation markers (sP-selectin) compared to those with COVID-19. (2) In patients with in-hospital CVE, proADM and endothelin were higher compared to those without in-hospital CVE, exhibiting higher levels in cases of CAP than in those of SARS-CoV-2 pneumonia. CitH3 was higher in those with CVE and CAP; however, statistical significance was not reached in those without CVE. (3) Higher significant levels of proADM and endothelin were associated with in-hospital mortality whereas CitH3 showed a non-significant trend, with patients with CAP presenting the most elevated levels. (4) ProADM and endothelin showed significant predictive value for one-year mortality in patients with CAP and SARS-CoV-2 pneumonia.

Endothelial damage, NETs, and platelet activation are among the pathophysiological mechanisms involved in CAP and COVID-19. Concerning exaggerated inflammation, prior studies have shown higher cytokine systemic levels in CAP compared to COVID-19, although other components of the pathophysiological pathways are not well-known [18,19,20]. Cytokines, such as IL-8, have been linked to endothelial damage, increased platelet activation, and the production of NETs [21,22,23,24]. A greater cytokine-mediated inflammatory response in CAP compared to COVID-19 could explain, at least in part, greater endothelial damage, platelet activation, and NET production in the first group.

In our study, we found a greater systemic response of endothelial dysfunction, NETosis, and platelet activation at day 1—as measured by biomarkers—mainly in CAP compared to COVID-19 from the first wave in Spain. These results have not been explored in other subsequent waves. Large studies have demonstrated that endothelial damage is a recognized, poor prognostic factor in both COVID-19 and CAP [25,26,27,28,29,30]. NETosis and platelet activation have also been associated with a worse prognosis in both diseases [13,14,15,31,32]. However, there is little information on shared and comparative endothelial damage in COVID-19 and CAP. In intensive care unit (ICU)-admitted patients, Bhatraju et al. compared different biomarkers of inflammation, endothelial, and epithelial damage in a case–control study. They analyzed 78 patients from a non-COVID-19 heterogeneous group including pneumonia and sepsis, as well as 93 patients with a COVID-19-confirmed diagnosis [33]. They found lower levels of markers of endothelial dysfunction (angiopoietin 2:1 ratio) and inflammation (soluble tumor necrosis factor receptor 1) in COVID-19 compared to patients without COVID-19. In another case–control study, Hokama et al. compared E-selectin, Von Willebrand factor, and tissue factor in patients with sepsis (n = 21) vs. mild (n = 31) or severe COVID-19 (n = 24), reporting significantly higher levels of E-selectin in sepsis compared to COVID-19 [34].

Concerning NETosis, particularly in CAP, higher levels of NETs were correlated with worse outcomes [13]. In COVID-19, higher levels of systemic NETs (H3Cit and elastase–DNA complex) were shown in more severe cases [35]. There are no studies concerning sP-selectin in a comparison between CAP and COVID-19. Investigators Karsli et al. evaluated sP-selectin in COVID-19 pneumonia and healthy controls. They found that sP-selectin is higher in severe COVID-19 pneumonia compared to mild and healthy controls [36].

After considering cardiovascular risk factors (hypertension, dyslipidemia, and diabetes) by matching, we observed that the frequency of CVE was higher in CAP compared to COVID-19 except for pulmonary embolism (PE). Despite this, the CAP cohort had a higher median age and more chronic heart disease. These factors may have influenced this outcome. Several studies in hospitalized patients with COVID-19 reported higher CVE (25–35%) compared to previous studies in CAP, although it should be noted that a large number of CVEs in COVID-19 are due to PE [37,38,39]. Our study is the first case–control analysis which compares not only CVE but also host response in COVID-19 vs. CAP.

Interestingly, we confirm that in patients with CVE, endothelial marker levels were higher in COVID-19 and CAP compared to those without CVE. The most elevated levels observed were those in CAP. These results may explain why patients with CAP suffer more long-term CVE (13.2% vs. 2.0%, *p* = 0.003). Previous literature suggests a potential link between NETs and CVE in COVID-19 as well [40]. However, as it relates to CitH3 and cfDNA biomarkers, we observed a trend for higher levels in those with CVE; the difference was not, however, statistically significant. Other studies have analyzed NETosis in lung tissue finding higher concentrations in severe episodes.

As expected, patients with COVID-19 had higher in-hospital mortality (around 30%) than patients with CAP (5–10%). This is consistent with the pre-vaccination era with scarce antivirals [41,42,43,44]. In our study, higher levels of proADM and endothelin were found in patients who presented in-hospital and one-year mortality, regardless of whether they had COVID-19 or CAP. This is consistent with previous literature that reported more endothelial dysfunction in more severe episodes, resulting in organ damage and poorer outcomes [45,46]. In CAP, Menéndez et al. found that endothelial markers were higher in patients with worse prognoses at day 1 and later during evolution at day 30 [47]. In COVID-19, endothelialitis and endothelial damage have been reported in the pulmonary vasculature [48]. Higher endothelial damage biomarkers (ProADM and proendothelin) showed greater predictive mortality value compared to C-reactive protein, procalcitonin, and D-dimer [26,49]. Elevated levels of proADM (>1.76 nmol/L) or proendothelin (>75 nmol/L) were associated with a higher rate of ICU admission, in-hospital mortality, and shorter time to death [26], as seen in other series of cases [50,51]. Notably, initial levels of CitH3, cfDNA, and sP-selectin were not associated with higher mortality. Nevertheless, our study was conceived to evaluate potential differences between COVID-19 and CAP and not to identify differences related to mortality.

In our study, CitH3 was superior in CAP compared to COVID-19 patients, but it is not related to mortality. This is a similar finding to those present in the current literature. Other authors have suggested that it is also possible that NETs are sequestered in the damaged organs of patients, and circulating NETs do not correlate well with lung disease [15]. Ebrahimi et al. measured cell-free nucleosomes in patients with CAP, capable of correlating with worse outcomes [13]. However, the quantification of nucleosomes as NETosis markers may be overestimated due to a plausible fragmentation. Investigators Morimont et al. performed an observational study to evaluate the formation of NETs between ICU-admitted patients with septic shock (n = 48) and critical patients with COVID-19 (n = 22). Controls were matched by age, gender, and comorbidity (n = 48) [52]. They found that Nu.H3.1 (nucleosome marker) was higher in COVID-19 whereas neutrophil elastase (NE) showed the opposite trend. The remaining NET biomarkers did not show statistically significant differences. Levels of Nu.H3.1 increased with higher SOFA and APACHE-II scores in septic patients; however, an inverse correlation was observed for APACHE-II scores and Nu.H3.1 levels in COVID-19. Due to this finding, the authors concluded that Nu.H3.1 reflected distinct, potential pathological processes in acute respiratory distress syndrome (ARDS) conditions of this kind.

Interestingly, in the long term (1-year mortality), patients with CAP surviving the acute episode have significantly higher mortality rates compared to those with COVID-19. According to available literature, CAP is a risk factor for long-term mortality [44]. Mortality per year in CAP per previous studies is around 10%, a similar value to the mortality in our study (7.3%) [53]. An important fact is that many of these deaths are of cardiovascular origin, even in patients without known prior heart disease [54,55]. In COVID-19, mortality per year is around 3.8%—very similar to our mortality results (3.9%) [56]. Notably, both proADM and endothelin maintained statistical significance when predicting 1-year mortality after discharge. Finally, as previously mentioned, a close relationship between endothelial damage and the inflammatory response has been established. Hence, the use of these biomarkers of endothelial damage could be useful in making clinical decisions on corticosteroid prescription. However, previous observational studies have demonstrated the ability of corticosteroids to reduce the host’s inflammatory response but not endothelial damage [57]. Therefore, clinical trials are required to evaluate the usefulness of corticosteroids and other drugs in reducing endothelial damage in COVID-19.

Some limitations of our study must be acknowledged: (1) cfDNA may originate or be released from different sources apart from NETs; (2) biomarker levels were only evaluated in ED samples, and no serial samples were obtained; (3) despite matching, baseline characteristics show more COPD and more chronic heart disease in patients with CAP; (4) although there is a considerable number of patients, the sample size is limited with regard to mortality and some complications; (5) the patients with COVID-19 were selected at the beginning of the pandemic, so these findings could be different if patients from other “waves” (e.g., delta) were selected.

## 4. Materials and Methods

### 4.1. Study Design and Participation

We conducted a case–control study in hospitalized patients with COVID-19 and CAP at La Fe University and Polytechnic Hospital in Valencia, Spain. We matched patients by age, sex, dyslipidemia, arterial hypertension, diabetes, and SpO_2_/FiO_2_ in the emergency department (ED). The Biomedical Research Ethics Committee Hospital La Fe approved the study (2020-114-1).

Diagnosis of pneumonia required compatible signs and clinical symptoms and a new radiological infiltrate. Exclusion criteria were residence in a nursing home, age under 18 years, admission in the previous 15 days, immunosuppressive status, and refusal of written informed consent. COVID-19 diagnosis was confirmed by reverse transcription polymerase chain reaction (RT-PCR) testing of a nasopharyngeal swab or sputum samples.

Patients with COVID-19 were recruited between March and April 2020. Patients with CAP were matched from an initial cohort of 1115 patients enrolled between February 2012 and November 2019 (2013/0204). Additionally, fifty healthy controls from a historical cohort were analyzed (Supplemental Table S2).

Demographic data, comorbidity, initial severity, complementary explorations, treatments, and 1-year follow-up were recorded in a data protocol. Evaluated outcomes included in-hospital and 1-year follow-up mortality and cardiovascular complications. Cardiovascular complications were previously defined [25].

### 4.2. Blood Samples

Samples were obtained at ED visits or during the first morning after admission (initial 12 h after ED arrival). Peripheral venous blood was drawn from patients and kept in ethylenediaminetetraacetic acid (EDTA) tubes. Hemolyzed blood samples were rejected. EDTA tubes were centrifugated (2500 rpm) for 10 min to obtain plasma and aliquoted for storage at −80 °C until examination. NETs, platelet activation, and endothelial injury were evaluated using the plasma samples. To improve measurement precision, all biomarker analyses were performed twice. The mean for both determinations was obtained.

### 4.3. Neutrophil Extracellular Traps

Detection of NETs was assessed with cfDNA and CitH3, considered one of the most specific markers of NET formation [58]. As found in previous studies, NETs can predict more severe episodes of COVID-19 pneumonia and estimate different clinical trajectories [59].

Plasma samples were mixed with a monoclonal mouse anti-histone biotinylated antibody (Component 1, Cell Death Detection ELISAPLUS) in a streptavidin-coated plate (Component 9). A rabbit polyclonal anti-histone-H3 (citrullinated R17 + R2 + R8) (ab81797; Abcam Inc., Waltham, MA, USA) antibody was used in a second step. Detection was carried out with a peroxidase-linked antibody (GE Biosciences, Barcelona, Spain). A pool of samples from normal subjects was used to normalize values. It was included in all microplates, being expressed as individual absorption values.

To determine cfDNA, plasma was diluted 1:10 with phosphate-buffered saline (PBS (in mmol/L: NaCl 137, KCl 2.7, Na_2_HPO_4_ 10, KH_2_PO_4_, pH 7.4)) and mixed with an equal volume of 1 mm SytoxGreen (Invitrogen, Carlsbad, CA, USA). Fluorescence was determined in a fluorescence microplate reader (Gemini XPS; Molecular Devices, Sunnyvale, CA, USA). A calibration curve was generated with calf thymus DNA (Invitrogen) in PBS. More detailed information is provided elsewhere [60].

### 4.4. Platelet Activation

sP-selectin was measured as a documented platelet activation marker, which is associated with inflammation, endothelial damage, and neutrophil activation [61]. Determination of sP-selectin was carried out with commercially available enzyme-linked immunosorbent assay (ELISA) kits from Affymetrix (eBioscience, Horsham, UK). Plasma was diluted 1:10 with a sample diluent. Absorbance readings were performed with a spectrophotometer using 450 nm; concentration (ng/mL) was calculated with a standard curve for human sP-selectin ELISA, obtained according to the manufacturer’s instructions. It was included in all microplates.

### 4.5. Endothelial Damage

Endothelin and proADM were measured by immunofluorescent assays according to the manufacturer’s instructions (Thermo Scientific BRAHMS through TRACE technology in KRYPTOR Compact Plus, Horsham, UK). The same reagent kit lots were used on all samples. More details on the measurement of these biomarkers are provided elsewhere [26].

### 4.6. Clinical Outcomes: Definitions

The occurrence of in-hospital or follow-up cardiovascular events was considered if acute coronary syndrome, new or worsening congestive heart failure, new or recurrent arrhythmia, or stroke appeared [25]. Pulmonary embolism was considered if there was CT scan evidence of a thrombus. Mortality was recorded at three periods: in the hospital, at 30 days, and at 1 year. Mortality due to any reason was also recorded. More details on clinical outcomes are available elsewhere [25].

### 4.7. Statistical Analysis

The statistical analysis was performed using IBM SPSS Statics version 26.0 software. COVID-19 and CAP data were matched using the MatchIt package, which implements suggestions made by Ho et al. [17,62]. The “Optimal” method was used to find the matched samples with the smallest average absolute distance across all the matched pairs. Age, sex, dyslipidemia, arterial hypertension, diabetes, and SpO_2_/FiO_2_ at ED were used for matching. A standardized mean difference of 0.181 was obtained.

Baseline characteristics and follow-up outcomes and complications were collected. Data were summarized as median (1st quartile, 3rd quartile) or count (%) for continuous or categorical variables, respectively. Statistical significance was considered if the *p*-value ˂ 0.05.

For the comparison of biomarker levels and analytical parameters between COVID-19 pneumonia and CAP, a Mann–Whitney U test was used according to the non-normality of the sample. For the comparison of baseline characteristics, complications, and other qualitative variables, a chi-square test with N-1 Campbell correction was performed to correct the low frequency of some categories.

Univariate associations are expressed as unadjusted OR with 95% confidence intervals (CIs).

## 5. Conclusions

Our findings provide relevant information showing more endothelial damage, NET production, and platelet activation in CAP compared with COVID-19 pneumonia. In the short term, there are worse consequences in patients with COVID-19; however, in the long term, the worst outcomes occur in those with CAP. ProADM and endothelin are associated with in-hospital mortality, in-hospital CVE, and 1-year mortality from admission.

## Figures and Tables

**Table 1:** Baseline characteristics, severity, and respiratory support.

	COVID-19 Pneumonia (n = 179)	CAP (n = 179)
Age, years, median (1st quartile, 3rd quartile)	65 (53, 79)	70 (56, 79)
Male sex, no. (%)	105 (58.7)	108 (60.3)
Current or former smokers, no. (%)	48 (26.8)	141 (78.8)
Coexisting conditions, no. (%)		
Hypertension	78 (43.6)	86 (48.0)
Diabetes	45 (25.1)	47 (26.7)
Dyslipidemia	57 (31.8)	71 (39.7)
Overweight *	102 (57.0)	69 (38.6)
COPD	4 (2.2)	42 (23.5)
Asthma	8 (4.5)	24 (13.4)
Chronic heart disease	23 (12.9)	61 (34.1)
Chronic renal disease	27 (15.1)	26 (14.5)
Neurological disease	27 (15.1)	21 (11.7)
SpO_2_/FiO_2_ at admission (1st quartile, 3rd quartile)	452.4 (435.7, 457.1)	442.9 (419.1, 457.1)
Radiological data at admission		
Bilateral infiltrates, no. (%)	111 (62.0)	33 (18.4)
Maximum respiratory support, no. (%)		
No respiratory support	79 (44.1)	66 (36.9)
O_2_ nasal cannula	18 (10.1)	78 (43.6)
O_2_ venturi/reservoir mask	56 (31.3)	22 (12.3)
HFNC	9 (5.0)	2 (1.1)
CPAP/NIMV	2 (1.1)	7 (3.9)
MV	15 (8.4)	4 (2.2)
Etiology, no. (%)	NA	102 (57)
Bacterial	NA	88 (49.2)
*Streptococcus pneumoniae*	NA	38 (21.2)
Atypical	NA	36 (20.1)
Viral	NA	25 (14)
Influenza	NA	19 (10.6)
Mixed (bacterial and viral)	NA	11 (6.1)

SpO_2_/FiO_2_: peripheral blood oxygen saturation/fraction of inspired oxygen; HFNC: high-flow nasal cannula; CPAP/NIMV: continuous positive airway pressure/non-invasive mechanical ventilation; MV: mechanical ventilation; NA: not applicable. * Body mass index ≥ 25.

**Table 2:** Comparison of in-hospital and total 1-year mortality and complications.

	COVID-19 Pneumonia (n = 179)	CAP (n = 179)	*p*-Value
In-hospital outcomes			
Mortality, no. (%)	26 (14.5)	7 (3.9)	0.001
Cardiac complications, no. (%)	11 (6.2)	18 (10.1)	0.176
Acute coronary syndrome, no. (%)	3 (1.7)	0 (0.0)	0.082
Arrhythmia, no. (%)	5 (2.8)	9 (5.0)	0.276
Congestive heart failure, no. (%)	4 (2.2)	11 (6.2)	0.065
Pulmonary embolism, no. (%)	9 (5.0)	0 (0.0)	0.002
Stroke, no. (%)	0 (0.0)	2 (1.1)	0.156
Total 1-year outcomes			
Mortality, no. (%)	33 (18.4)	20 (11.2)	0.053
Cardiac complications, no. (%)	14 (7.8)	35 (19.6)	0.001
Acute coronary syndrome, no. (%)	3 (1.7)	5 (2.8)	0.475
Arrhythmia, no. (%)	6 (3.4)	18 (10.1)	0.011
Congestive heart failure, no. (%)	6 (3.4)	21 (11.7)	0.003
Pulmonary embolism, no. (%)	9 (5.0)	0 (0.0)	0.002
Stroke, no. (%)	0 (0.0)	5 (2.8)	0.024

**Table 3:** Mortality and complications at 1-year follow-up after discharge (in survivors).

	COVID-19 Pneumonia (n = 153)	CAP (n = 172)	*p*-Value
1-year follow-up mortality, no. (%)	7 (4.6)	13 (7.6)	0.001
1-year cardiovascular complications, no. (%)	3 (2.0)	23 (13.4)	0.003
1-year acute coronary syndrome, no. (%)	0 (0.0)	5 (2.9)	0.082
1-year arrhythmia, no. (%)	1 (0.7)	13 (7.6)	0.005
1-year congestive heart failure, no. (%)	2 (1.3)	14 (8.1)	0.009
1-year pulmonary embolism, no. (%)	0 (0.0)	0 (0.0)	NA
1-year stroke, no. (%)	0 (0.0)	3 (1.7)	0.287

NA: not applicable.

## Data Availability

The datasets used and/or analyzed during the current study are available from the corresponding author upon reasonable request.

## Supplementary Materials

The following supporting information can be downloaded at: https://www.mdpi.com/article/10.3390/ijms241713194/s1.
