# Supplementary material for: Endothelial Damage, Neutrophil Extracellular Traps and Platelet Activation in COVID-19 vs. Community-Acquired Pneumonia: A Case–Control Study
Source: Int J Mol Sci. 2023 Aug 25;24(17):13194. doi: 10.3390/ijms241713194 (PMC10488034; doi:10.3390/ijms241713194)
Supplement: Supplementary file 1 [file ijms-24-13194-s001.zip › ijms-2567733-supplementary.pdf]

SUPPLEMENTAL FILE

Table S1: ***Baseline characteristics, severity and respiratory support in initial CAP cohort.***

	Initial CAP cohort (n=1115)
Age, years, median (1st quartile, 3rd quartile)	*72 (59,81)*
Male sex, no. (%)	*693 (62.2)*
Current or former smokers, no. (%)	*650 (59.9)*
Coexisting conditions, no. (%)	
HTA	*591 (53.2)*
Diabetes	*308 (27.6)*
Dyslipidemia	*233 (42.7)*
*Overweight**	*327 (29.5)*
COPD	*228 (20.5)*
Asthma	*86 (7.7)*
Chronic heart disease	*373 (33.5)*
Chronic renal disease	*149 (13.4)*
Neurological disease	*185 (16.6)*
SpO2/FiO2 at admission, median (1st quartile, 3rd quartile)	*442.9 (417.6, 456.7)*
Bilateral infiltrates at admission, no. (%)	*203 (18.2)*
Maximum respiratory support, no. (%)	
No respiratory support	*434 (39.3)*
O2 nasal cannula	*442 (40)*
O2 venturi/reservoir mask	*155 (14)*
HFNC/CPAP/NIMV	*48 (4.3)*
MV	*25 (2.3)*

**Body mass index ≥25.*

Table S2: ***Characteristics of healthy controls.***

	Healthy controls (n=50)
Age, years, median (1st quartile, 3rd quartile)	*43 (40,49)*
Male sex, no. (%)	*17 (34)*
Current or former smokers, no. (%)	*5 (10)*
Coexisting conditions, no. (%)	
HTA	*3 (6)*
Diabetes	*0 (0)*
Dyslipidemia	*4 (8)*
*Overweight**	*NA*
COPD	*0 (0)*
Asthma	*1 (2)*
Chronic heart disease	*1 (2)*
Chronic renal disease	*0 (0)*
Neurological disease	*0 (0)*

*NA: not available. *Body mass index ≥25*
